# Superoxide dismutase and the sigma1 receptor as key elements of the antioxidant system in human gastrointestinal tract cancers

**DOI:** 10.1515/biol-2021-0124

**Published:** 2021-11-24

**Authors:** Michał Skrzycki

**Affiliations:** Chair and Department of Biochemistry, Warsaw Medical University, 02-097 Warsaw, Banacha 1, Poland

**Keywords:** superoxide dismutase, sigma1 receptor, gastrointestinal cancer, oxidative stress, antioxidant enzymes

## Abstract

This long-term research was designed to evaluate whether superoxide dismutase (SOD) isoenzymes participate in the development of human gastrointestinal neoplasms and the potential influence of the sigma1 receptor (Sig1R) on the regulation of *SOD* gene expression during the neoplastic process. The experiments included human tissues from selected gastrointestinal tract tumors (liver cancer, colorectal adenocarcinoma, and colorectal cancer liver metastases). Activity, protein levels, and mRNA levels were determined for SOD isoenzymes and Sig1R. Additionally, markers of oxidative stress (glutathione, lipid peroxidation) were measured. The results showed significant changes in the antioxidant system activity in all examined types of tumors. SOD changed both in healthy cells and in neoplastic cells. The activity and expression of all studied enzymes significantly changed due to the advancement of tumor development. The Sig1R might be an additional regulator of the antioxidant system on which activity might depend on the survival and proliferation of cancer cells. Overall, the study shows that SOD1 and SOD2 are involved not only in the formation of neoplastic changes in the human gastrointestinal tissues (healthy intestine – colon tumor; healthy liver – liver cirrhosis – liver cancer) but also in the development of tumors in the sequence: benign tumor – malignant tumor – metastasis.

## 1 Introduction

The current state of knowledge clearly indicates that oxidative stress is one of the most important causes of the formation and development of gastrointestinal neoplasms. Inflammation and external environmental factors (food antigens, bacteria, viruses, fungi, parasites, and toxins), metabolism of ethanol and xenobiotics, and excess iron in the diet favor the formation of large amounts of free radicals and reactive forms of oxygen and nitrogen (ROS, RNS) in the membrane cells of the intestine, stomach, pancreas, and liver. As a result, the damage of proteins, lipids, and DNA might lead to neoplastic transformation [1,2,3,4].

The first ROS resulting from the single-electron reduction of oxygen is the superoxide anion radical (O_2_^−^). It undergoes a dismutation reaction to hydrogen peroxide (H_2_O_2_), which might be converted into a hydroxyl radical (HO*) in a Fenton or Haber–Weiss reaction. Further reactions lead to the formation of less common ROS and reactive forms of nitrogen (e.g., peroxynitrite NOO–). All the above-mentioned compounds strongly react with the molecular components of the cell, but in lower concentrations, they can act as intracellular messengers [5,6].

### 1.1 Oxidative stress

The disparity between the formation and inactivation of reactive oxygen species leads to oxidative stress. A long-term state of oxidative stress causes disturbances in cellular metabolism, which are related to the damage caused by the action of ROS. This increases the risk of irreversible changes in cellular macromolecules such as proteins, lipids, DNA, and carbohydrates. The loss of activity of some proteins, changes in the fluidity of lipid membranes, mutations, and damage to nucleic acids are the most common consequences of ROS action in normal and neoplastic cells [7,8].

In addition to the activity of antioxidant enzymes, the most significant markers of oxidative stress in the body are the level of lipid peroxidation, the concentration of glutathione (GSH), carbonyl groups, and 8-hydroxy-2'-deoxyguanosine. Lipid peroxidation is a chain of reactions in cell membranes under the influence of O_2_^−^ and/or HO*. The end products of this process (e.g., malondialdehyde [MDA], 4-hydroxynonenal) are highly reactive compounds that can last in cells longer than reactive oxygen species and the ability to diffuse from where they are formed to distant regions of the cell.

GSH is the most common, low molecular weight antioxidant. The GSH/GSH from oxidized glutathione (GSSG) redox balance is sustained by the activity of antioxidant (GSHPx, glutathione *S*-transferase [GST]) and complementary (glutathione reductase [GSHR]) enzymes in its concentration. GSH and its dependent enzymes play a key role in the detoxification of peroxides, hydroperoxides, xenobiotics, and drugs [9].

### 1.2 Superoxide dismutase (SOD) as the component of the antioxidative system

The amount of ROS present in the cell depends on the activity of both the enzymes that generate them and the enzymes removing them, that is, antioxidant enzymes. Small-molecule antioxidants also play an important role in passive ROS inactivation, for example, GSH, tocopherols, vitamins C and A, and others [10,11].

Numerous enzymes are involved in removing ROS from the cell. The SOD is the first and key antioxidant enzyme responsible for the dismutation reaction of the superoxide radical anion to H_2_O_2_. In humans, there are three SOD isoenzymes: cytoplasmic (SOD1/CuZnSOD – copper–zinc), mitochondrial (SOD2/MnSOD – manganese), and extracellular (ECSOD) [12,13,14].

The product of the SOD reaction – H_2_O_2_ is decomposed in two ways: at high concentrations by catalase (CAT) and at lower concentrations by selenium-dependent glutathione peroxidase. Both reactions lead to the formation of water but glutathione peroxidase uses GSH as a cofactor for the reaction. This enzyme belongs to the group of GSH-dependent enzymes. GSH-dependent enzymes also include selenium-independent glutathione peroxidase (GSHPx), which removes organic peroxides. In turn, GST couples toxins and xenobiotics with GSH, whereas GSHR is responsible for the renewal of GSSG. The excess of transition metals catalyzing the Fenton and Haber–Weiss reactions is removed by chelating proteins called metallothioneins [15,16,17,18].

### 1.3 Sigma1 receptor (SigR) as the regulatory element of the antioxidative system

The Sig1R is a membrane and cytosol protein with a sequence similar to fungal C-8.7 sterol isomerase but without any other known homology with other mammalian proteins. The *SIGMAR* (Sigma1 receptor) gene is located on human chromosome 9p13. This gene is expressed as a single 30 kDa polypeptide containing 223 amino acids. Sig1R is located in the plasma membrane or the endoplasmic reticulum (ER) due to the presence of two transmembrane domains [19,20]. The Sig1R might be involved in the pathogenesis of many diseases (neurodegenerative, depression, addiction, arthritis, Crohn’s disease, and tumors), but there is not enough data to fully elucidate its function.

The Sig1R is most often placed between the ER membrane and the mitochondrion, where it binds to the IP3 receptors and ankyrin. The Sig1R is involved in numerous processes:

– regulating the ion channel function for calcium, potassium, sodium, and chlorine [21,22,23],
– inhibiting the nitric oxide synthase activation [24],
– acting as a chaperone protein – might be a cell stress receptor [25], and
– influencing apoptosis and cell proliferation by regulating the functioning of signaling pathways for Akt and extracellular signal-regulated kinases (ERK) [26].

Moreover, it exhibits chaperone activity and translocation ability from the ER membrane to the cell membrane. As Sig1R appears to have a considerable effect on the levels of numerous ions needed by the cell, it influences significant cell metabolism pathways [27].

Numerous scientific studies reported an increased expression of the Sig1R in various types of cancer (breast, prostate, lung, melanoma, and glioma), which might be necessary for regulating SOD isoenzymes. Increased level of Sig1R changes the functioning of the Akt and ERK pathways. The Akt pathway activates the nuclear factor kappa-light-chain-enhancer of activated B cells (NF-kB) transcription factor, and the ERK pathway activates the c-Fos transcription factor (AP-1 subunit). Both of these factors directly participate in the regulation of transcription of all SOD isoenzymes. In addition, the activity of the Sig1R might change depending on the presence of ROS in the cell, and it might directly affect the amount of NF-kB protein [28,29].

The goal of this study was to analyze multiple data from a number of previous researches done in our department. Such complex analysis and collation of different data allowed for new and comprehensive conclusions concerning the activity of the antioxidant system during tumor development.

## 2 Materials and methods

### 2.1 Sample collection

The study involved 166 people aged 23–80 years operated on due to gastrointestinal cancer. The mean age of the patients was 54.5 ± 14.5 years. The study group consisted of 10 patients with gastric cancer, 13 patients with adenocarcinoma, 25 with cirrhosis and 30 with liver tumors, 65 with primary colorectal cancer, and 33 with metachronous colorectal cancer liver metastases. All studied types of neoplasms had a surgical and histopathological diagnosis.

Among liver tumors, benign tumors (adenomas (*n*-3), benign adenomas (*n*-3), hemangiomas (*n*-4)) and malignant neoplasms – hepatocellular carcinoma (*n*-17) and choloangiocarcinoma (*n*-3) – were diagnosed. Primary colorectal cancers studied were diagnosed as adenocarcinomas (Table 1).

**Table 1:** Clinicopathological data of studied subjects

Clinical diagnosis	Mean age	Women	Man	Sum
Liver cirrhosis	33.3 ± 8.9	11	14	25
Hepatic cancer	46.8 ± 13.6	13	17	30
Primary colorectal cancer (adenocarcinoma)	65.8 ± 11.4	30	35	65
Colorectal cancer to liver metastases	58.4 ± 10.8	14	19	33
	Sum	68	85	153

Patients with colorectal cancer liver metastases underwent chemotherapy and radiotherapy before the surgery. The research material consisted of tissue and blood samples collected from patients (with their consent) operated in 2002–2005 in the Department of General, Transplant and Liver Surgery; Department of General, Gastroenterological and Nutrition Surgery and Department of General and Transplant Surgery, Medical University in Warsaw. The control tissue was a section taken 6–7 cm from the tumor border during surgery.

Blood was collected from patients 1 day before surgery and 6 days after surgery (if possible). The control group consisted of blood collected at the Blood Donation Station from 53 healthy donors: 32 women and 21 men (average age: 36 years) not treated for cancer.


**Informed consent:** Informed consent has been obtained from all individuals included in this study.
**Ethical approval:** The research related to human use has been complied with all the relevant national regulations, institutional policies and in accordance with the tenets of the Helsinki Declaration, and has been approved by the Bioethics Committee at the Medical University of Warsaw (decision KB/164/2001 from 09.10.2001).

### 2.2 Preparation of tissue extracts

Immediately after surgery, the tumor section and control tissue were washed in 0.9% NaCl and frozen at −70°C. Tissues were cut into smaller pieces, weighted, and homogenized (five times at 1 min intervals with 3 min breaks) in nine volumes of chilled 100 mM Tris-HCl buffer (pH 7.5) containing 5 mM MnCl_2_, 100 mM KCl, and 1% (v/v) Triton X-100 using a Heidolph Diax 600 brand homogenizer (Sigma-Aldrich) at 9,500–13,000 rpm. After 30 min extraction on a magnetic stirrer, the homogenates were frozen at −70°C. After 24 h of freezing, they were centrifuged at 12,000*g* for 30 min at 4°C. The obtained tissue extracts (supernatants) were used to determine the activity of SOD isoenzymes and the expression of CuZnSOD (SOD1), MnSOD (SOD2), AP-1, and NF-kB at the protein level. Blood sera and prepared tissue extracts were stored at −70°C until they were used for research.

### 2.3 Cell lines

Experiments with altered oxygen levels were performed on the human SW480 (primary colon adenocarcinoma) and SW620 (lymph node metastases from the same patient) cell lines. Cell lines were provided by American Type Culture Collection – Virginia, USA. Both cell lines were grown under standard conditions in a Dulbecco’s modified Eagle medium (DMEM) in a 37°C/5% CO_2_ humidified incubator. The medium was supplemented with 10% fetal bovine serum, penicillin (100 U/mL), streptomycin (100 µg/mL), and HEPES (20 mM). After 5 days of culture, cells were harvested by treatment with 0.25% trypsin–0.02% ethylenediaminetetraacetic acid (EDTA) in phosphate-buffered saline (PBS) and used for experiments.

To determine the effect of oxygen, cells (4 × 10^4^) were seeded in DMEM and grown under hypoxia (1% O_2_), normoxia (10% O_2_), and atmospheric normoxia (21% O_2_) in a hypoxic chamber with an oxygen controller (Coy Laboratory Products Inc., Grass Lake, MI). The viable cell number was assessed with tryptophan blue dye. Exclusion test performed by an automatic cell counter (Countess Invitrogen, Waltham, MA).

### 2.4 Determination of SOD1 and SOD2 activity

#### 2.4.1 SOD1

The method of determination relies on the production of a superoxide anion by the reaction of xanthine with xanthine oxidase (XOD), which, by reacting with 2-(4-iodophenyl)-3(4-nitrophenyl)-5-phenyltetrazole (INT) chloride, forms a red formazan dye. The measurement of the absorbance of the colored product determines the effectiveness of the above reaction.

\text{Xantine}\hspace{.25em}\mathop{\longrightarrow }\limits^{\text{XOD}}\hspace{.25em}\text{Uric}\hspace{.25em}\text{acid}\hspace{.25em}+{{\text{O}}_{2}}^{-}]

\text{I}.\text{N}.\text{T}.\hspace{.25em}\mathop{\longrightarrow }\limits^{{{\text{O}}_{2}}^{-}}\hspace{.25em}\text{Formazan}\hspace{.5em}\text{dye}]

CuZnSOD presented in the tested sample (biological material) inhibits this reaction by dismutating O_2_^−^ to H_2_O_2_ and O_2_. The degree of inhibition of this reaction is directly proportional to the activity of CuZnSOD.

{{\text{O}}_{2}}^{-}\hspace{.25em}+\hspace{.25em}{{\text{O}}_{2}}^{-}\hspace{.25em}+\hspace{.25em}2{\text{H}}^{+}\hspace{.5em}\mathop{\longrightarrow }\limits^{\text{CuZnSOD}}\hspace{.3em}{\text{O}}_{2}\hspace{.25em}+\hspace{.25em}{\text{H}}_{2}{\text{O}}_{2}]

CuZnSOD activity was determined spectrophotometrically using a standard RANSOD kit by Randox (United Kingdom), which includes the following reagents:

1. Substrate: Xanthine 0.05 mmol/L, INT 0.025 mmol/L;
2. Buffer: N-cyclohexyl-3-aminopropanesulfonic acid 40 mmol/L, pH 10.2, EDTA 0.94 mmol/L;
3. Xanthine oxidase 80 U/mL; and
4. Standard – CuZnSOD 5.5 U/mL.

CuZnSOD activity in the tested biological material was determined in a Shimadzu UV 1202 spectrophotometer (Kyoto, Japan), measuring the absorbance at 0 and 3 min at a wavelength of 505 nm.

#### 2.4.2 SOD2

MnSOD activity was determined spectrophotometrically according to the method described by Beauchamp and Fridovich [30] and modified by Oberley and Spitz [31]. The method is based on the production of a superoxide radical by the reaction of xanthine with XOD. The resulting superoxide anion reduces nitrotetrazole blue chloride –  2,2'-di-*p*-nitrophenyl-5,5'-diphenyl-3,3'-[3,3'-dimethoxy-4,4'-diphenylene] – chloride ditetrazole (NBT) to form a color complex. Measurement of the absorbance of the color product determines the effectiveness of the above reaction.

CuZnSOD activity in this reaction is inhibited by adding 0.33 M NaCN.

\text{Xantine}\hspace{.3em}\mathop{\longrightarrow }\limits^{\text{XOD}}\hspace{.3em}\text{Uric}\hspace{.25em}\text{acid}\hspace{.05em}+{\text{O}}_{2}]

\text{NBT}\hspace{.3em}\mathop{\longrightarrow }\limits^{{{\text{O}}_{\text{2}}}^{-}}\hspace{.3em}\text{Color}\hspace{.25em}\text{product}]

The degree of inhibition of this reaction is directly proportional to the activity of MnSOD.

{{\text{O}}_{2}}^{-}\hspace{.25em}+\hspace{.25em}{{\text{O}}_{2}}^{-}\hspace{.25em}+\hspace{.25em}2{\text{H}}^{+}\hspace{.3em}\mathop{\longrightarrow }\limits^{\text{MnSOD}}\hspace{.25em}{\text{O}}_{2}\hspace{.25em}+\hspace{.25em}{\text{H}}_{2}{\text{O}}_{2}]

For the determination of MnSOD activity in the biological material tested, a reaction mixture was prepared containing the following components: 1.33 mM *N*,*N*-bis[2-(bis[carboxymethyl]amino)ethyl] glycine pentenoic acid, CAT (1 U/mL), 2.25 mM NBT, 1.8 mM xanthine, and 0.33 M NaCN dissolved in 0.05 M phosphate buffer, pH 7.8.

The mixture with the test sample was incubated for 30 min at room temperature. A XOD (80 U/mL) was then added to initiate the O_2_^−^ production reaction.

MnSOD activity in the tested biological material was determined in a Shimadzu UV 1202 spectrophotometer by measuring the absorbance at time 0 and after 2 min at a wavelength of 560 nm.

The protein concentration in the tissue extracts of the tested samples was determined by the Bradford method [32].

CuZnSOD and MnSOD activities were expressed in units per milligram protein. CuZnSOD activity in blood serum was expressed in units per milliliter of serum.

### 2.5 Determination of lipid peroxidation

The level of lipid peroxidation was determined by measuring the end products of lipid peroxidation that react with thiobarbituric acid (thiobarbituric acid reactive substances – TBARS) [33]. The pink chromogen resulting from the reaction of thiobarbituric acid with MDA and other secondary lipid peroxidation products was estimated at 532 nm. The results are expressed as nanomoles of TBARS per liter of blood serum.

### 2.6 Measurement of GSH level

Reduced GSH level was measured in blood serum as described by Sedlak and Lindsay, and Snel et al. [34,35]. The method uses the formation of the colored product, which is formed by the reaction of GSH with 5,5′-dithio-bis-[2-nitrobenzenic acid] (DTNB). The amount of GSH–DTNB conjugate was determined by the change in absorbance at 412 nm. GSH level was expressed as micromoles per liter of blood serum [36].

### 2.7 Quantitative determination of SOD isoenzymes protein level

The immunodetection method – Western blotting – was used to quantify the expression of SOD1 and SOD2 proteins in studied tissues [34]. The tissue extracts were prepared for electrophoresis by sonification and heating in Leamli buffer. Next, vertical electrophoresis was carried out on a 6% thickening gel and a 14% separation gel containing sodium dodecyl sulfate according to the Laemmli method, using BioRad equipment. After 50 min of semidry electrotransfer, the immunoblotting was carried out. Primary polyclonal antibodies (Calbiochem, Merck group) used in the study were diluted in the following ratio: 1:2,000 for SOD1 and 1:500 for SOD2. Detection of studied proteins was performed with the “ECL plus western blotting analysis kit” from Amersham Life Science. The exposure time of Kodak Bio-MAX MR film was 2, 5, and 10 min [37]. To confirm an equal amount of protein in the gel, after removing primary and secondary antibodies, the membrane was reincubated with anti-actin antibodies (Santa-Cruz company). The analysis was performed in a UVI – KS 4000 I densitometer camera, using Scion – Image and ZERO – DSCAN programs.

### 2.8 Determination of SOD isoenzymes and Sig1R gene expression at mRNA level with RT-PCR

The total RNA of the samples was isolated by the TRIzol method according to the protocol provided by the manufacturer. cDNA was amplified by reverse transcriptase - polimerase chain reaction (RT-PCR). The mixture containing 1 µg of DNase-treated RNA and 0.25 mM oligo (dT) 15 primer (Sigma-Proligo, Sigma-Aldrich Group) was heated at 70°C for 5 min to denature secondary RNA structures and then cooled on ice. The RT-PCR reaction mixture containing 0.2 mM deoxy Nucleo Tri Phosphates (dNTP), 1 mM dithiothreitol, 2 U/µL RNase inhibitor, and 10 µL moloney murine leukemia virus reverse transcriptase (Fermentas, Thermo - Scientific Company) was incubated at 42°C for 60 min and then heated to 70°C for 10 min. The cycle was repeated 25 times.

To amplify the DNA of the *SIGMAR1* gene, the appropriate primers were synthesized: 5' AGCGCGAAGAGATAGC 3' (sense) and 5' AGCATAGGAGCGAAGAGT 3' (antisense). To amplify the DNA of the *SOD1* and *SOD2* genes, the appropriate primers are synthesized. A pair of primers for SOD1 had the following sequences: 5' CCTAGCGAGTTATGGCGACG 3' (meaningful) and 5' CAACATGCCTCTCTTCATCC 3' (antisense). A pair of primers for SOD2, the following: 5' AACCTCACATCAACGCGCAG 3' (meaningful) and 5' CCAACAGATGCAGCCGTCAG 3' (antisense). The volume of products received was 258 bp for SOD1 and 307 bp for SOD2.

The PCR reaction mixture contained 1.0 mM MgCl_2_, 1 µM primers, 0.2 mM dNTP, 1 µL Taq DNA polymerase. Initial denaturation was at 94°C for 3 min. Denaturation conditions during PCR were at 94°C for 1 min. The primer binding temperatures were 54.2°C for SIGMAR1, 54.2°C for SOD1, 56.4°C for SOD2, and 55°C for the β-2 microglobulin.

Elongation conditions were at 72°C for 2 min. A total of 30 cycles were used for each of the genes. Then the reaction mixture was heated to 72°C for 4 min and cooled to 4°C. The PCR reaction was performed in a PCT 200 DNA ENGINE thermocycler from MJ RESEARCH.

The mRNA level was determined densitometrically using the UVI – KS 4000 I camera and Scion – Image and ZERO – DSCAN programs. The optical density (OD) of the SOD isoenzymes and Sig1R cDNA was compared to that of the β-2 microglobulin cDNA, which allowed for the determination of semiquantitative values of the gene expression level (OD Sig1R or SODs/OD B2M) [38].

### 2.9 Statistical analysis

The obtained results were subjected to statistical analysis using the calculation of arithmetic means (*x*) with standard deviations using appropriate statistical tests. Two tests were used to compare the distributions of the variable in two groups: the parametric *t*-test and the parametric Wilcoxon test (also called the Mann–Whitney test). In addition, two measurement methods were used to assess the significance of the correlation: the classical Pearson’s correlation coefficient and the nonparametric Spearman’s correlation coefficient. ANOVA was used to compare the means of multiple groups. In the assessment of the statistical significance of differences between the means, the significance level was *p* < 0.05.

## 3 Results and discussion

In the studies on the antioxidant barrier status in gastrointestinal neoplasms (benign and malignant liver cancer, colorectal adenocarcinoma, metastasis of colorectal cancer to the liver), a significantly intensified lipid peroxidation and disturbances in GSH levels and GSH-dependent enzymes activity were found in patient’s serum. The highest level of lipid peroxidation was observed in gastric cancer, whereas the lowest level characterized liver cancer. Lipid peroxidation was also increased in colorectal cancer and its metastases to the liver. Results of this research point to the oxidative stress accompanying the formation and development of gastrointestinal neoplasms. An important result of these studies is the demonstration that increased peroxidation of lipids in the blood serum of patients was characteristic for all studied neoplasms of the gastrointestinal tract. No such changes in the level of GSH and the activity of GSH-dependent enzymes were found. It is not clear whether the systemic disorders of GSH red/ox in subjects with gastrointestinal neoplasms might be the causative agent of cancer or if the neoplastic disease itself is a cause of such disorder (Table 2) [39].

**Table 2:** Oxidative stress markers (GSH and lipid peroxidation) in blood serum of patients with various types of gastrointestinal tract cancers

Clinical diagnosis	Healthy donors	Hepatic cancer		Colorectal cancer		Colorectal cancer to liver metastases	
Blood serum	Control	Before surgery	After surgery	Before surgery	After sugery	Before surgery	After sugery
*N*	30	11	10	11	12	11	14
TBARS nmol/L	1.2 ± 0.2	2.2 ± 0.4*	1.5 ± 0.2**	3.1 ± 0.5*	2.8 ± 0.5*	3.5 ± 0.3*	1.7 ± 0.1**
GSH µmol/L	15.2 ± 4.1	18 ± 4.6	12.1 ± 0.9	18.5 ± 2.4	16 ± 2.1	37.3 ± 10.3*	34 ± 10.6*

TBARS (lipid peroxidation) level in blood serum of patients with gastrointestinal tract tumors. * Statistically significant versus control serum (*p* < 0.05). ** Statistically significant versus serum before surgery (*p* < 0.05). GSH level in blood serum of patients with gastrointestinal tract tumors. * Statistically significant versus control serum (*p* < 0.05). ** Statistically significant versus serum before surgery (*p* < 0.05).

Because the adaptation of neoplastic cells to oxidative stress relies on modifying their own antioxidant systems [40,41,42], the activity and expression of antioxidant enzymes (in particular SOD – the key antioxidant enzyme) at various stages of cancer development were tested. First, the level of reduced GSH and the activity of GSH-dependent enzymes (GSHPx, GST, and GSHR) in neoplastic tissues originating from various types of gastrointestinal neoplasms were analyzed. Significant changes in the activity of GSH-dependent enzymes and the level of GSH have been observed in gastric cancer, benign and malignant liver cancer hepatocellular carcinoma, primary colorectal cancer, and colorectal cancer liver metastases [43].

Our earlier research results indicate a disturbance of the antioxidant system based on GSH and GSH-dependent enzymes in tumors of the gastrointestinal tract. That is probably due to the generation of large amounts of ROS in tumor cells. Changes in the level of GSH and the activity of GSH-dependent enzymes in gastrointestinal neoplasms are connected with the altered ability to detoxify ROS, which might act as an adaptation mechanism of neoplastic cells to oxidative stress conditions.

However, the most important enzymes defending cells against oxidative stress are SOD isoenzymes – it depends on them how many and what free radicals and reactive oxygen species will be formed from the superoxide radical anion [44].

Due to the fact that superoxide anion is a precursor of the remaining ROS and RNS, studies were conducted to determine changes in the activity and level of the protein of SOD isoenzymes (SOD1/CuZnSOD and SOD2/MnSOD) in liver cancers. The applied research model in setup, the liver cirrhosis (precancerous stage) – benign tumor – malignant liver tumor, gave an answer to the question of whether the level of antioxidant enzymes depends on the extent of tumor development [45].

**Figure 1:**
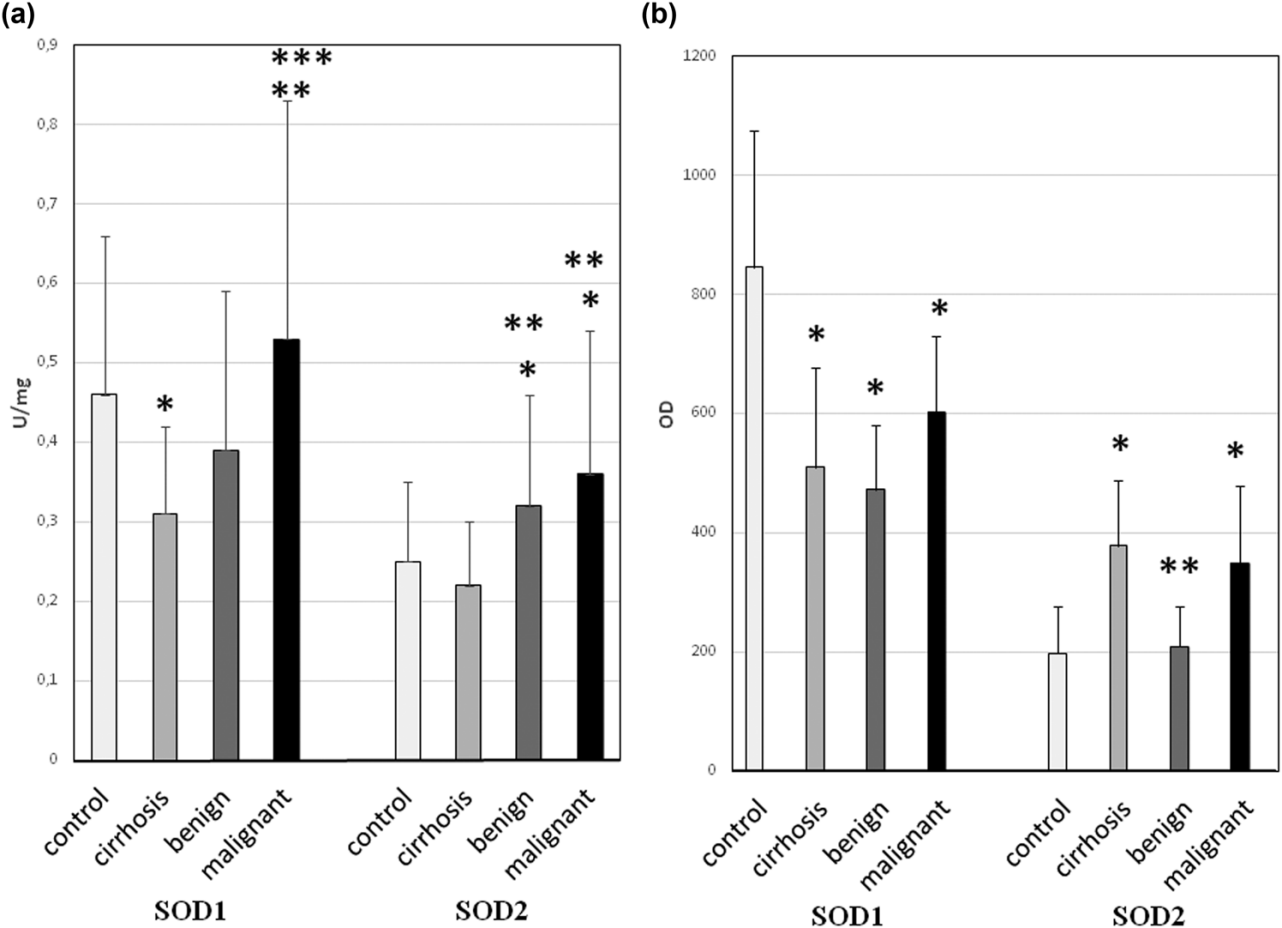
Activity and protein level of SOD isoenzymes in liver cirrhosis and liver tumors. Sample tissues were arranged due to increasing neoplasticity of liver cells, that is, cirrhosis (*n* = 25), benign tumors (*n* = 10), and malignant tumors (*n* = 20). Control was healthy tissue surrounding tumors. (a) Activity of SOD1 and SOD2 – activity of SOD isoenzymes was measured according to description in methods and expressed as units per milligram of protein. We observed the highest activity of both SODs in malignant tumors. However, there were also large error bars – it might be caused by variability of particular patients age and health, which influence total red/ox balance of organism. (b) Protein level of SOD1 and SOD2 – the amount of protein of SOD isoenzymes was measured by standard western blot and expressed as OD. The protein level does not exactly correspond with activity of SODs; still malignant tumors have higher protein level than benign ones. Discrepancy might be caused by partial inactivation of enzyme due to an increased presence of free radicals in tumor cells. Some large error bars might be a result of variability of particular patient’s age and health, which influence total red/ox balance of organism. * – statistically significant versus normal liver (*p* < 0.05), ** – statistically significant versus liver cirrhosis (*p* < 0.05), and *** – statistically significant versus benign liver tumors (*p* < 0.05).

The obtained results showed significant changes in SOD1 and SOD2 levels during the formation and development of liver tumors (Figure 1).

The activity of SOD1 and SOD2 varied depending on both the stage of the tumor and its cellular location. In the case of the cytoplasmic isoenzyme (SOD1), its activity was significantly reduced in the cirrhotic liver and benign liver tumors but increased in malignant ones. And in case of mitochondrial enzyme (SOD2), its activity did not change in the cirrhotic liver but was increased in both benign and malignant liver tumors. In all examined tissues except for benign liver tumors, SOD1 protein level was decreased, whereas SOD2 protein level increased. The low SOD activity in the cirrhotic liver leads to ROS accumulation, leading to oxidative damage in hepatocytes. That leads to a cascade of events starting with inflammation, further exacerbation of oxidative stress, protein damage caused by it, peroxidation of cell membrane phospholipids and mutations, followed by impaired functioning of genes and signaling pathways resulting in an increase in the rate of cell proliferation, and finally as a result of progressive damage to cellular structures for neoplastic transformation. The opposite situation in malignant liver cancer (increased activity and level of SOD protein) might indicate two-way effects: on the one hand, high activity of SOD causes the generation of large amounts of H_2_O_2_, which activates many signaling pathways (in particular those responsible for cell proliferation), whereas on the other hand, cancer cells with elevated SOD activity are more resistant to the oxidative stress associated with neoplastic disease. SOD protects cancer cells against the damage caused by ROS by sustaining the equilibrium among the elimination of O_2_^−^ and the formation of H_2_O_2_ while simultaneously providing the conditions for their increased proliferation. Consequently, instability of this equilibrium might determine whether cancer cells grow reluctantly and possibly apoptosis is activated or whether there would be an increase of proliferation, consecutively increasing their malignancy. The obtained outcome might point to the potential mechanism of malignant cancer cells bypassing oxidative stress effects, which permit their growth in a hostile environment (excess of reactive oxygen species).

In contrast to malignant tumors, in benign liver cancer, the compensatory effect of SOD isoenzymes (decreased SOD1 vs increased SOD2) was observed for the first time. This action allows the oxidant–antioxidant balance to be maintained in liver benign cancer cells. Cells with a balanced redox state are characterized by a low proliferation rate. This might be one of the causes why benign cancers develop slowly and are mild in their disease.

Assessment of the activity and protein expression of SOD isoenzymes between benign and malignant liver cancers illustrates an increase of both factors in malignant cancers. Increase of the activity and level of SOD1 and SOD2 proteins in malignant liver cancers above the level appropriate for normal cells implies improved resistance against oxidative stress in contrast to benign liver cancers. The obtained results for liver cancer indicate that various antioxidant mechanisms act more efficiently, particularly in malignant cancers. The tumor malignancy appears to depend on the cell redox status, which is largely regulated by SOD.

The conducted research has shown that the activity and level of SOD are associated with the degree of tumor development.

This was also confirmed by studies on the activity and level of SOD in colorectal cancer. It is a relatively well-described neoplasm with clearly classified stages of its clinical development and degrees of cell differentiation. Therefore, studies conducted on colorectal cancer allowed a detailed analysis of the antioxidant system permutations during the development of neoplastic disease. The activity and protein level of SOD isoenzymes and the level of lipid peroxidation (oxidative stress marker) were tested in primary colorectal cancer, which is distinguished by four clinical stages of cancer advancement (UICC – according to TNM classification) and three stages of maturity/differentiation of neoplastic cells (G) [46,47,48].

It has been shown that the activity and protein level of SOD isoenzymes changed depending on the clinical advancement stage and tumor maturity. The outcome of these researches showed that the initial stages of clinical advancement and differentiation of colorectal cancer are characterized by increased oxidative stress. However, the oxidative stress level was significantly lower in the subsequent stages of tumor development (UICC I–IV). It seems that oxidative stress in the early stages of cancer development might result from the inflammatory state related to neoplastic disease. Oxidative stress can be considered a kind of selective pressure – therefore, only cancer cells capable of adapting to it will pass into the next stages of development. This is indicated by the observed decreased level of lipid peroxidation in the following stages of colorectal cancer development.

Our research has shown that throughout the development of colorectal cancer, neoplastic cells have a constant level of antioxidant defense due to the cytosolic SOD activity.

**Figure 2:**
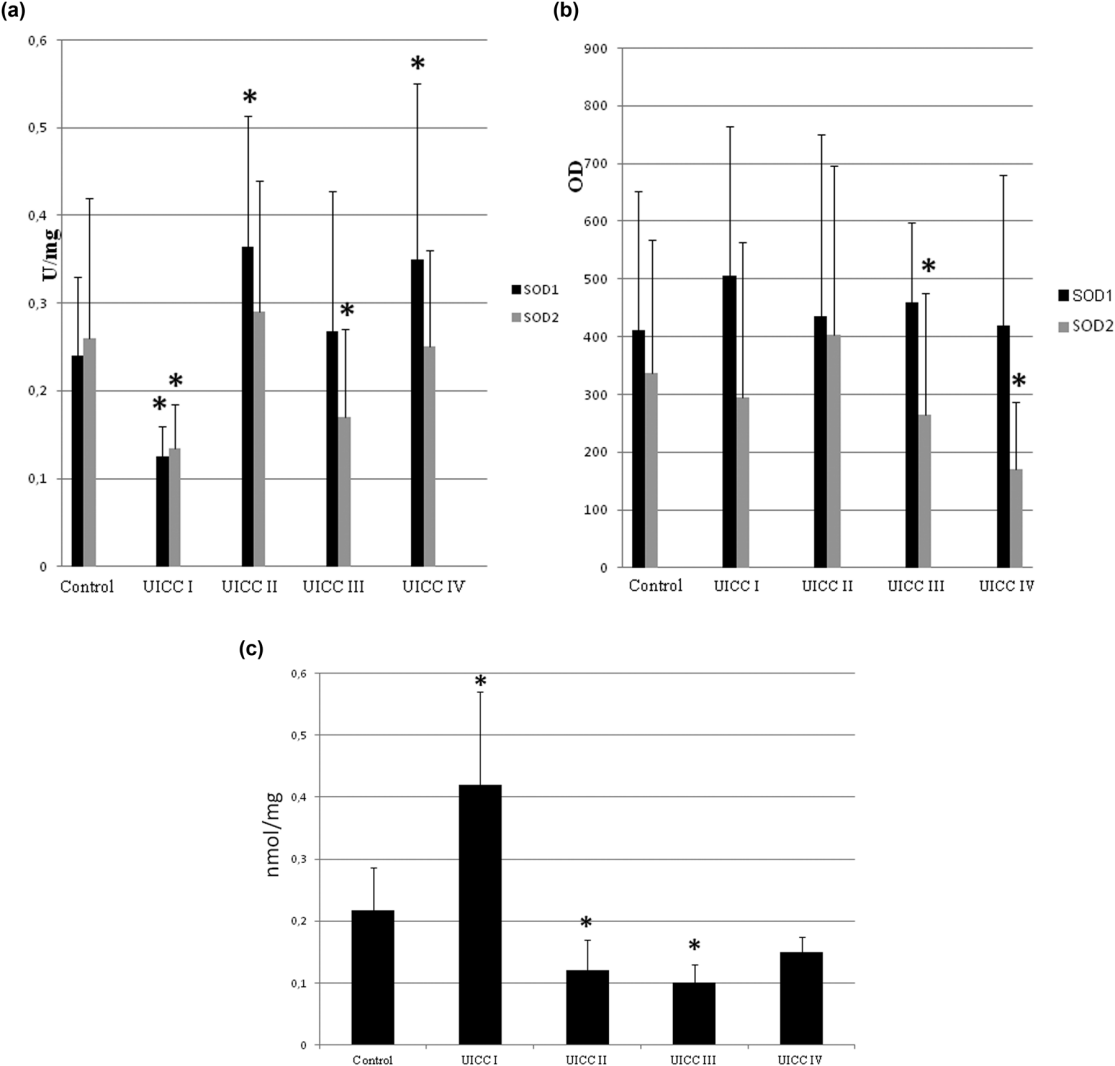
Lipid peroxidation, activity, and protein level of SOD isoenzymes in different stages (UICC) of colorectal cancer development. The UICC stages are divided to four and defined as follows: I – only single small tumors present, II – more than two tumors, III – larger tumors and nodes invaded, IV – all that was in previous stages plus metastasis observed. We ordered our colorectal cancer tissues in these stages using medical history of each subject. Number of cases: I = 5, II = 12, III = 9, and IV = 19 (we were able to identify the UICC stage only in part of studied subjects). (a) Activity of SOD1 and SOD2 – activity of SOD isoenzymes was measured according to description in methods and expressed as units per milligram of protein. (b) Protein level of SOD1 and SOD2 – the amount of protein of SOD isoenzymes was measured by standard western blot and expressed as OD. In general, we observed cyclic changes of both SOD activity and protein level dependent on UICC stage. It is more clearly visible for activity than for protein level of SOD isoenzymes. For detailed explanation, see text. (c) Lipid peroxidation level – its amount was measured and expressed as TBARS – thiobarbituric acid reactive substances in nanomoles per mg of protein. The highest lipid peroxidation level occurred in stage I, and then it is considerably low, but increases while cancer develops. Large error bars might be caused by small number of cases in each stage and differences between particular patient’s age and health. * – statistically significant versus healthy colon (*p* < 0.05).

In contrast, the mitochondrial enzyme SOD2 activity differed significantly in the following stages of the UICC but did not change due to the degree of differentiation. These results indicate that changes in SOD2 activity in the development of colorectal cancer are cyclical, that is, the activity decreased in stages I and III and increased in stages II and IV (comparing to control) (Figure 2).

An agent responsible for such fluctuations in SOD2 activity might be its reaction product – H_2_O_2_, which regulates SOD2 activity through feedback inhibition.

**Figure 3:**
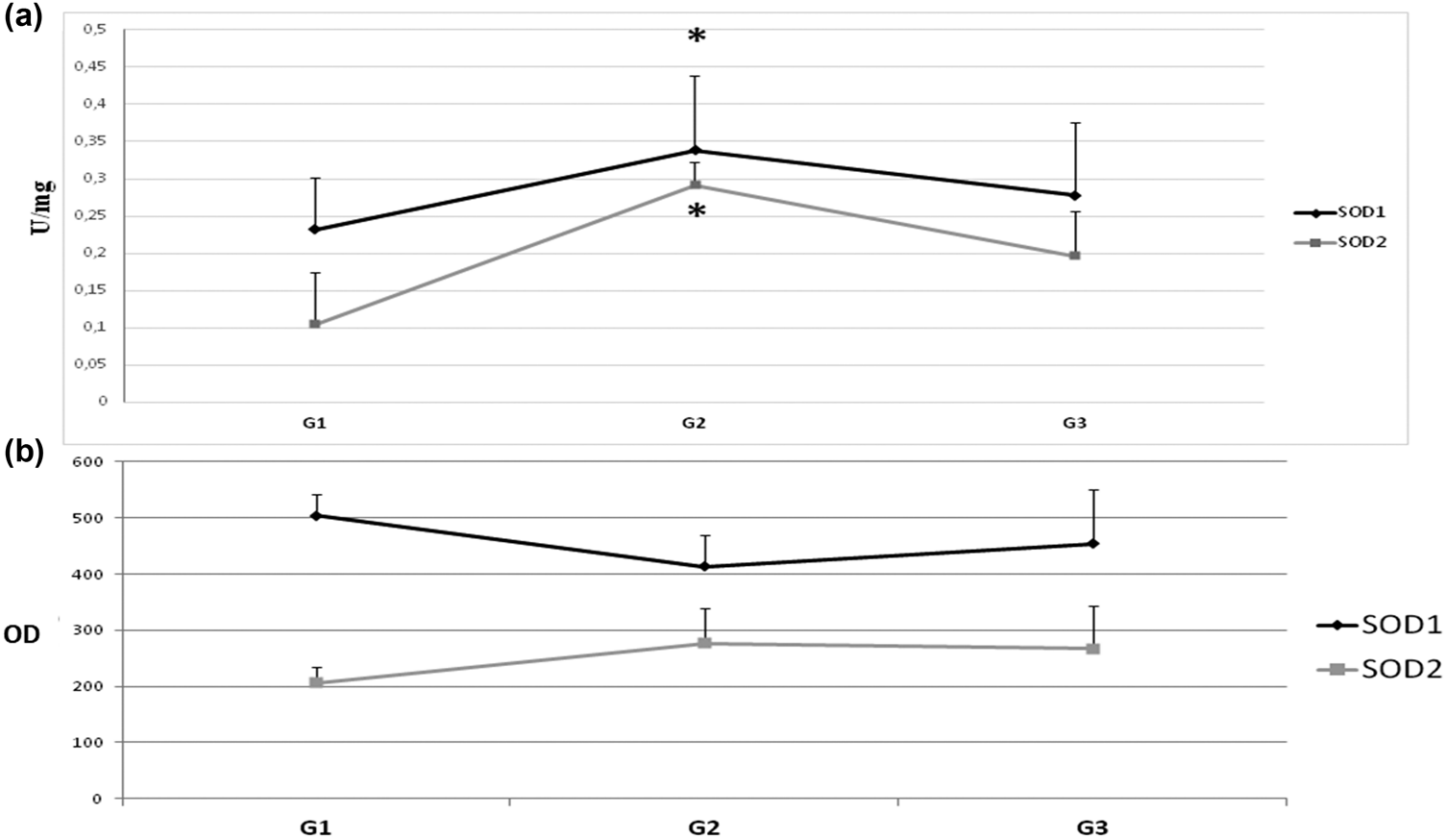
Activity and protein level of SOD isoenzymes in subsequent stages of tumor cell differentiation (G). To determine the maturity of the tumor, the classification G (grading) is used. According to the WHO criteria, there are three levels of cancer maturity: G1 – highly differentiated – cancer with histological and cytological features similar to normal glandular epithelium. G2 – moderately differentiated – a cancer of an intermediate structure. G3 – low-differentiated – cancer that does not have common histological features and cytology with normal glandular epithelium. Number of cases: G1 = 3, G2 = 24, and G3 = 11 (we were able to identify the G stage only in part of studied subjects). (a) Activity of SOD1 and SOD2 – activity of SOD isoenzymes was measured according to description in methods and expressed as units per milligram of protein. (b) Protein level of SOD1 and SOD2 – amount of protein of SOD isoenzymes was measured by standard Western blot and expressed as OD. We observed that both activity and protein level of SOD isoenzymes change in a cyclic manner due to differentiation stage (increase–decrease). The activity and protein level of SOD1 are clearly higher than those of SOD2. Activity is the highest at G2 stage. The protein level of SOD2 is also the highest in G2 stage, but in case of SOD1 it is the highest in G1 stage. * – statistically significant versus healthy colon (*p* < 0.05).

On the one hand, the level of the SOD2 protein in the subsequent stages of colorectal cancer differentiation also changed in a cyclical model: G1 – elevated, G2 – low, G3 – elevated again. On the other hand, SOD2 activity remained at a similar level. H_2_O_2_ appears to inhibit SOD1 as well. However, while the progression of colorectal cancer in subsequent stages of clinical advancement causes inflammation and oxidative stress due to intestinal wall overgrowth, differentiation is a change in cell histology and morphology and does not affect cell redox state. Therefore, changes in SOD protein level through differentiation might depend on the action of transcription factors (i.e., AP-1, NF-kB) and be only a secondary phenomenon that simultaneously controls the expression of SOD and contribute to the regulation of cell differentiation (Figure 3) [49].

The level of protein and the activity of SOD isoenzymes clearly changes in the successive stages of clinical advancement and differentiation degrees of colorectal cancer. These changes seem to depend primarily on the clinical stage of colorectal cancer. It is unclear whether the changes in SOD levels are correlated directly with cell differentiation. These results also indicate that changes in the protein level and activity of SOD isoenzymes might be an adaptive reaction to oxidative stress occurring in the course of the development of colorectal cancer.

The obtained results fully confirmed the assumptions of the working hypothesis, which allowed the scientific community to draw attention to the problem of changes in the expression and activity of antioxidant enzymes during the development of a neoplastic tumor.

Since the adaptive reaction to oxidative stress requires the activation of appropriate signaling pathways in cells, which will, in turn, activate the transcription of genes encoding antioxidant enzymes [36], another research model was developed to assess the expression of SOD isoenzymes both at the protein and mRNA levels in colorectal cancer [50].

Obtained results clearly show that the increased (comparing to control) level of SOD1 and SOD2 in the early stages of colorectal cancer development provides an effective defense against oxidative stress caused by inflammation. However a decreased protein level of the SOD isoenzymes in UICC stage III might indicate a destabilized antioxidant response of cancer cells, leading to further oxidation of cellular environment and, consequently, greater frequency of mutations. It would allow for changes in the phenotype of neoplastic cells required for the further development of cancer. In stage IV of UICC, differences in the levels of SOD1 and SOD2 proteins might indicate an attempt to reduce the level of ROS in the mitochondria to prevent apoptosis from being triggered, but at the same time to uphold the oxidative milieu in the cytoplasm required for proliferation and causing genetic instability.

**Figure 4:**
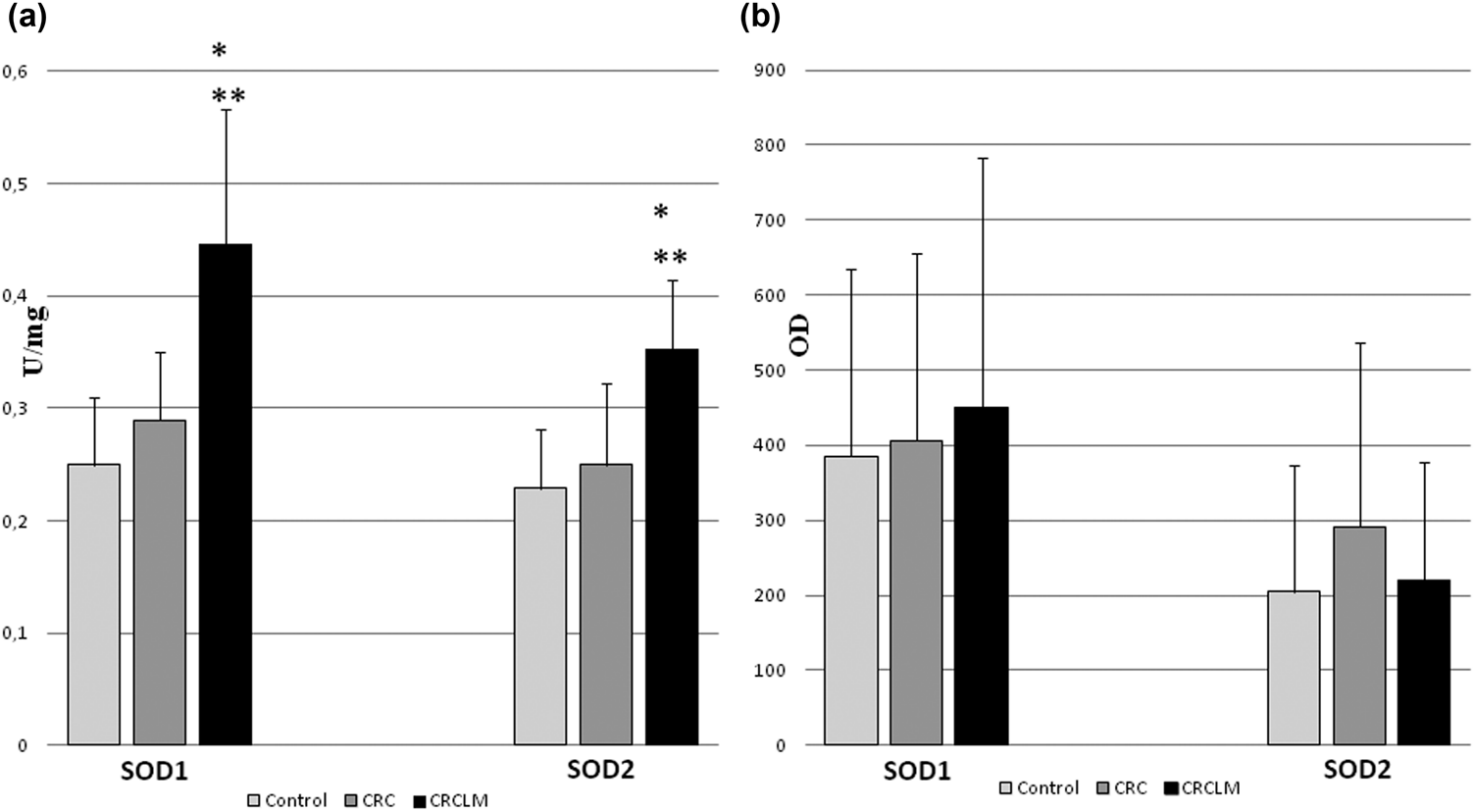
Activity and protein level of SOD isoenzymes in colorectal cancer and its liver metastases. CRC – colorectal cancer (*n* = 65); CRCLM – colorectal cancer liver metastases (*n* = 33); and control (*n* = 65). (a) Activity of SOD1 and SOD2 in CRC and CRCLM. Activity of SOD isoenzymes was measured according to description in methods and expressed as units per milligram of protein. We observed the highest activity of SODs in metastases comparing both to control and colorectal cancer. (b) Protein level of SOD1 and SOD2 in CRC and CRCLM. Amount of protein of SOD isoenzymes was measured by standard Western blot and expressed as OD. The highest level of protein was observed for SOD1 in metastases, whereas for SOD2 in colorectal cancer comparing to control. Large error bars might be a result of variability of particular patients age and health, which influence total red/ox balance of organism. * – statistically significant versus healthy colon (*p* < 0.05). ** – statistically significant versus colorectal cancer (*p* < 0.05).

The final stage of malignant cancer development is metastasis. The study also included subjects with metachronous metastases of colorectal cancer to the liver. The observed protein level of SOD isoenzymes clearly indicates the adaptation of cancer cells originating from the primary colon tumor to the liver environment. Liver metabolism generates large amounts of ROS as a result of cytochrome P450 activity. Therefore, cancer cells inhabiting the liver must protect their cytoplasm, which is validated by the high level of SOD1 observed in metastases (Figure 4).

The results acquired for the mRNA level of SOD isoenzymes in all clinical stages of colorectal cancer development indicate its inverse relationship to the protein level of SOD isoenzymes. The explanation for this discrepancy might be the regulation of SOD isoenzymes at the posttranscriptional level [51]. According to the literature data, an elevated mRNA level might point to a posttranscriptional reduction of the SOD protein level and activation of transcription by oxidative stress [52,53]. The differences between the mRNA and protein levels of SOD isoenzymes might depend on alterations of the red/ox cell status under the circumstances such as hypoxia, anoxia, and oxidative stress (each of these conditions occurs during tumor development).

The conducted studies indicate that the gene expression of SOD might be controlled on at least two levels: transcriptional and posttranscriptional. In contrast, the intracellular activation seems to be different for each of these levels, depending on endogenous cell conditions. SOD isoenzymes take part in maintaining the equilibrium between the generation and removal of ROS and might be one of the key enzymes determining apoptosis or survival of the cancer cell population.

Seemingly, determination of only SOD isoenzyme activity/protein level might not be sufficient to fully assess the adaptive potential of neoplastic cells to oxidative stress. Increased mRNA level might act as an unknown potential, capable of generating an additional amount of the enzyme in response to oxidative stress, whereas a decreased mRNA level of SOD might be a response to an elevated concentration of H_2_O_2_ to limit its pro-apoptotic effects.

The research performed in our department has shown that neoplastic cells adapt to oxidative stress by regulating the level of protein and mRNA of SODs. However, neoplastic cells maintain an increased ROS level required for their continued proliferation and play a significant role in providing an environment favoring genetic instability. For this purpose, the synthesis and activity of SOD isoenzymes must be regulated extremely precisely and on a multilevel basis.

The factor that might significantly affect the regulation of SOD isoenzymes expression seems to be the intracellular Sig1R. Recent scientific reports indicate its crucial role in cell metabolism [54,55,56].

So far, the relationship between the Sig1R and the level of SOD isoenzymes has not been studied. Therefore, we have carried out the study to clarify whether changes in Sig1R levels will be somehow related to oxidative stress and the transcription level of SOD isoenzyme genes in gastrointestinal cancers.

The first phase of the research investigated how Sig1R expression changes in colorectal cancer. The preliminary results of studies on the determination of the expression level of Sig1R in colorectal cancer and liver metastases at various stages of cancer progression are consistent with the literature data: increased levels of Sig1R were observed in colorectal cancer and also in metastases. Nevertheless, this was the only information available on this subject at that time (2013) in the literature data.

Sig1R expression level studies were performed for the first time, including clinical tumor advancement (UICC), tumor location in particular sections of the intestine, and patient age [57]. The highest expression level of the Sig1R was observed in the third stage of UICC. The ANOVA test also showed significant interactions between the clinical stage of the tumor and tumor location and the level of Sig1R expression. There was no interaction between the level of Sig1R mRNA, UICC grade, and the age of patients. However, a significant decrease in Sig1R expression was observed in older patients. It seems that the clinical stage, tumor location in the intestine, and age significantly influence the expression of the Sig1R. This is probably due to the two-fold nature of this receptor: under certain conditions, it can act pro- or antiapoptotic agent. Such effect might be due to the expression-dependent Sig1R activity.

**Figure 5:**
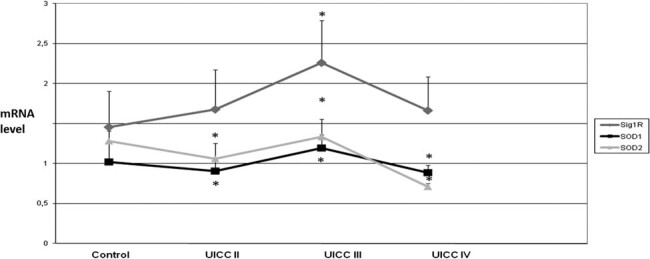
The mRNA level of SOD isoenzymes and Sig1R in different stages (UICC) of colorectal cancer development. The expression of SOD isoenzymes and Sig1R genes was determined by PCR and showed as OD of β-microglobulin vs OD of tested genes yielding semiquantitative units. It was unable to isolate mRNA from stage I colorectal cancer tissues. Nevertheless, we still observed a cyclic changes of SOD expression level dependent on UICC stage, and Sig1R expression seems to change in similar way. * – statistically significant versus healthy colon (*p* < 0.05).

**Figure 6:**
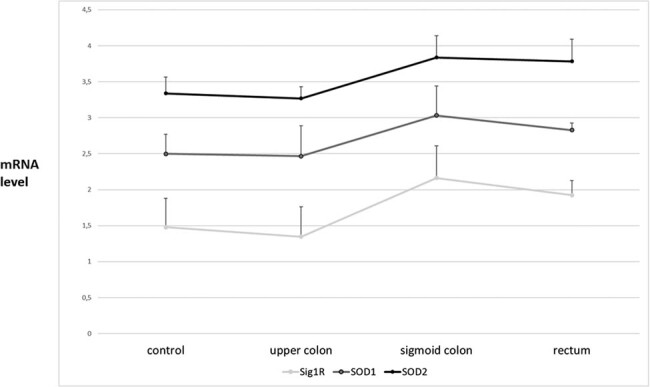
The mRNA level of SOD isoenzymes and Sig1R in various sections of the intestine. Control – respective sections of intestine from nontumorous tissue. Upper colon (*n* = 13), sigmoid colon (*n* = 22), and rectum (*n* = 10) – sections of tumors localized there. We studied if potentially different red/ox conditions in selected sections of intestine could influence on expression of SOD isoenzymes and Sig1R. There are noticeable differences of SODs and Sig1R expression dependent on localization in intestine. However, there were significant only in sigmoid colon and rectum, comparing to control. * – statistically significant versus healthy colon (*p* < 0.05).

Comparative statistical analysis of the results concerning the expression of the SOD isoenzymes and the Sig1R showed that the expression profile of all tested proteins (SigR1, SOD1, and SOD2) significantly depends on the tumor development stage (UICC) and is significantly different in each section of the intestine. This might indicate a relationship between the expression of these three proteins in cancer (unpublished data) (Figures 5 and 6).

The above observations open up a completely new perspective of research on the relationship of the Sig1R, reactive oxygen species, and antioxidant enzymes (SOD) in the formation and development of cancer.

In previous studies, we described subsequent studies carried out to evaluate the expression of Sig1R and SOD isoenzymes in the *in vitro* experimental model. We used different oxygen concentrations occurring during tumor development – hypoxia, tissue normoxia, and atmospheric normoxia (most of the studies on cell lines are conducted in these conditions) [58].

Diversified oxygen availability significantly influenced the expression level of all examined parameters, especially SOD2 and Sig1R. The type of cell line studied (primary cancer, metastatic cells) also influenced mainly the expression of Sig1R [59].

**Figure 7:**
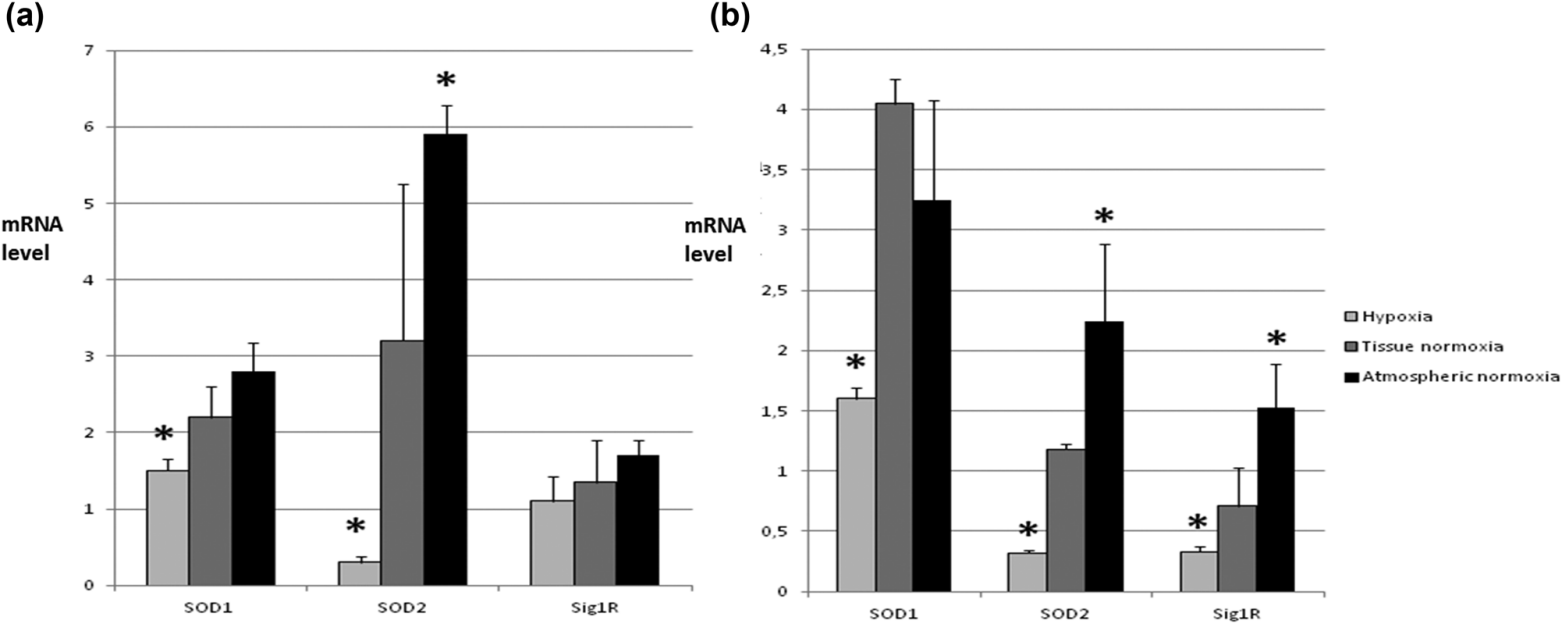
Expression of SOD isoenzymes and Sig1R in different oxygen concentrations. Three different concentrations of oxygen were chosen corresponding to hypoxia (below 1% O_2_ – condition often occurring during tumor growth; tissue normoxia (10% O_2_) – normal concentration of oxygen in healthy tissues; and atmospheric normoxia (21% O_2_) – often the concentration of oxygen under which cell lines are grown *in vitro*. (a) mRNA level of SOD1, SOD2, and Sig1R in SW480 cells. Here we observe that tested parameters change depending on oxygen concentration in the same manner – increasing while O_2_ increase. The most dramatic change is for SOD2. (b) mRNA level of SOD1, SOD2, and Sig1R in SW620 cells. Tested parameters change in very similar way like in SW480 cells; however, the general expression level of SOD isoenzymes is lower and Sig1R comparable to SW480 cells. * – statistically significant versus tissue normoxia (*p* < 0.05).

Statistical analysis revealed a strong relationship between the level of Sig1R, SOD2 expression, and cell survival under hypoxic conditions (1% oxygen). This points to the protective role of Sig1R and SOD2 in mitochondria. It is related to the regulation of the calcium ions’ cellular concentration and their influx to the mitochondria through ion channels regulated by Sig1R and oxidative stress (Figure 7).

The obtained results indicate the role of mitochondria as the main organelle enabling cancer cells to exist in conditions of oxidative stress and hypoxia. For the first time, due to the applied research model, our results show how oxygen conditions, such as those in the environment of a neoplastic tumor, affect the mechanisms that protect cells against oxidative stress.

The latest literature data confirms the Sig1R effect on the reactive oxygen species generation [60]. It was observed that compounds that are agonists of this receptor, by affecting the activity of the first respiratory chain complex, increased the level of ROS. The effect of Sig1R on this complex was calcium dependent. The induction of ROS secretion by the Sig1R might be associated with changes in the activity or expression of SOD isoenzymes in reaction to the elevated level of ROS in the cell [61].

Not only the activity but also the level of the Sig1R protein influences the activity of the respiratory chain. Some studies indicate that the knockout of the *SIGMAR1* gene caused increased production of ROS in cancer cells, and in CHO cells, additionally induction of the NF-kB transcription factor (one of the inducers of SOD2 expression) [62].

Other authors have shown that Sig1R can stimulate the expression of the so-called antioxidant response element and the expression of cytoplasmic SOD (SOD1) [25,63].

The results and literature data obtained in the course of the research confirm direct and indirect relationships between the Sig1R and SOD isoenzymes.

## 4 Conclusions

The antioxidant barrier is an important element of the body’s overall protection against the development of cancer. The effects of increased oxidative stress might have a long-term impact on the functioning of many organs, especially those that are particularly vulnerable to it, such as the gastrointestinal tract. Our research on gastrointestinal tract tumors has contributed to the understanding and clarification of many issues related to the functioning and regulation of key antioxidant enzymes. The obtained results clearly show that:

1. Oxidative stress accompanies the neoplastic disease
  + occurred in all examined types of gastrointestinal tract tumors
  + it is systemic (serum, tissues)
  + caused changes in the antioxidant enzymes activity, especially SOD, and in the level of small molecule antioxidants (GSH).
2. Activity and protein level of SOD, the key antioxidant enzyme, varied depending on location in the cell (SOD1 and SOD2) and the type of tumor (benign – malignant), as well as in the precancerous stage (cirrhosis).
3. The expression of the SOD isoenzymes (SOD1 and SOD2) changes with the successive stages of tumor development. This correlation occurs both at the stage of transcription and translation of *SOD* genes.
4. The expression of SOD isoenzymes is influenced by both oxidative stress and the advancement of the neoplastic changes.
5. The gene expression level of SOD isoenzymes is important for the adaptation of neoplastic cells to oxidative stress and, consequently, for the progression of neoplastic disease.
6. The Sig1R (stress–response protein) might affect the expression of SOD isoenzymes at subsequent stages of tumor development (UICC) and in various sections of the intestine (colon, sigmoid colon, rectum).
7. Changes in the expression of SOD isoenzymes and Sig1R indicate mitochondria as the main cell compartment allowing neoplastic cells to adapt to oxidative stress.
